# Combination of White Blood Cell Count at Presentation With Molecular Response at 3 Months Better Predicts Deep Molecular Responses to Imatinib in Newly Diagnosed Chronic-Phase Chronic Myeloid Leukemia Patients

**DOI:** 10.1097/MD.0000000000002486

**Published:** 2016-01-15

**Authors:** Ya-Zhen Qin, Qian Jiang, Hao Jiang, Yue-Yun Lai, Hong-Hu Zhu, Yan-Rong Liu, Bin Jiang, Xiao-Jun Huang

**Affiliations:** From the Peking University People's Hospital, Peking University Institute of Hematology, Beijing Key Laboratory of Hematopoietic Stem Cell Transplantation, Beijing, China.

## Abstract

The aim of this study was to evaluate the impact of white blood cell (WBC) counts at presentation on the achievement of deep molecular response.

A total of 362 newly diagnosed chronic-phase chronic myeloid leukemia patients (CML-CP) receiving 400 mg/day imatinib were serially monitored for a median of 36 months (range 6–115).

Patients showing an optimal response at 3, 6, and 12 months as defined by the 2013 European LeukemiaNet recommendations had significantly lower WBC counts at presentation than those showing nonoptimal responses (all *P* < 0.0001). Among the cutoff values with a similar Youden index, 150 × 10E9/L (abbreviated WBC > 150) was selected to identify the greatest amount of patients with the potential to achieve a sustained molecular response of 4.5 (MR4.5). Regardless of whether the Sokal risk score was included, the BCR-ABL^IS^ value at 3 months, WBC counts at presentation, hemoglobin levels, and sex were the common independent predictors for an MR4.5, with the former 2 presenting the highest hazard risk. Low Sokal risk scores did not independently predict the achievement of an MR4.5. Patients with concurrent WBC > 150 and BCR-ABL^IS^ ≤ 10% had a similar incidence of 4-year MR4.5 compared with patients with concurrent WBC ≤ 150 and BCR-ABL^IS^ > 10% and concurrent WBC > 150 and BCR-ABL^IS^ > 10% (13.5% vs 13.2% vs 8.8%, *P* = 0.47), and all of these values were significantly lower than the values for patients with concurrent WBC ≤ 150 and BCR-ABL^IS^ ≤ 10% (55.0%, all *P* < 0.0001). Patients with concurrent WBC ≤ 150 and BCR-ABL^IS^ ≤ 10% had better 4-year event-free survival rates, progression-free survival rates, and overall survival rates compared with patients with WBC > 150 or BCR-ABL^IS^ > 10%.

The combination of WBC count at presentation and BCR-ABL^IS^ at 3 months provides improved predictions of deep molecular response in imatinib-treated CML-CP patients. Therefore, the WBC count at presentation might be used to differentiate patients at the beginning of imatinib treatment.

## INTRODUCTION

The introduction of the tyrosine kinase inhibitor (TKI) greatly revolutionized the treatment of chronic myeloid leukemia (CML), and most patients can currently achieve a life expectancy similar to that of the general population.^1–5^ To improve the quality of life of patients and decrease the economic burden, “treatment-free remission” (TFR) has been proposed in recent years.^6,7^ Accordingly, rapidly achieving a deep molecular response has become the present treatment goal because a sustained molecular response of 4.5 (MR4.5) is linked to long-term outcomes ^8,9^ and the general enrollment criterion for discontinuation in clinical trials.^10,11^ Although second-generation TKIs have been reported to achieve significantly deeper and faster responses for first-line treatment compared with that of imatinib,^12,13^ certain side effects are more frequent and severe.^14,15^ Therefore, stratifying patients according to their response is required to guide treatment choice.

According to the international scale (BCR-ABL^IS^), BCR-ABL transcript levels > 10% at 3 months are associated with reduced rates of event-free survival (EFS), progression-free survival (PFS), and overall survival (OS).^16–18^ Furthermore, Branford et al demonstrated that the BCR-ABL^IS^ at 3 months independently predicted stable and undetectable BCR-ABL.^19^ At present, the failure to generate an early molecular response (EMR) to imatinib is a widely used criterion for switching to a second-generation TKI in chronic-phase CML (CML-CP).^2,20^

In addition to the response indicators during treatment, the parameters at diagnosis reflect biological characteristics of the patients and have a prognostic value. In CML, the Sokal, Euro, and EUTOS risk scores are generally used for stratification at presentation. Of note, these systems were developed to predict survival rates and cytogenetic responses but not deep molecular responses.^21–23^ Therefore, although the prognostic value of these systems has been demonstrated for certain levels of molecular response in a number of studies,^24–27^ these systems might provide incomplete assessments; thus, the parameters at presentation must be evaluated comprehensively.

To date, few studies have explicitly evaluated the prognostic value of white blood cell (WBC) counts at presentation in CML on the patient's response and outcome, although the lack of such evaluations might be related to high WBC counts presenting as a common feature of CML. However, a high WBC count is a strong high-risk pretreatment parameter in acute leukemia.^28,29^ During the establishment of Sokal risk scores, WBC counts were prognostic for the univariate analyses but insignificant for the multivariable regressions for survival. Recently, Hughes et al showed that patients presenting with early molecular response (EMR) failure had higher median WBC counts at the study start,^18^ which implied that WBC counts might be relevant to molecular responses. In addition, certain reports have demonstrated or implied that a combination of parameters at presentation with EMR might provide more accurate predictions of patient response and outcome.^20,27^ In this study, 362 newly diagnosed imatinib-treated CML-CP patients were investigated, and we showed that both a WBC count ≤ 150 × 10E9/L at presentation and BCR-ABL^IS^ > 10% at 3 months independently predicted an MR4.5. Furthermore, the combination of these parameters better predicted the achievement of an MR4.5 compared with the use of BCR-ABL^IS^ at 3 months alone.

## METHODS

### Patients and Treatment

A total of 362 patients were included in this study, and they were all diagnosed with CML-CP between April 2005 and June 2014 and received 400 mg/day imatinib within 6 months of diagnosis except during instances of severe toxicity. These patients were treated for at least 6 months and serially monitored at our center. The patient characteristics at presentation are shown in Table 1. The median time from diagnosis to the commencement of imatinib treatment was 1 month (range 0–6). During treatment, 14 patients switched to Nilotinib or Dasatinib because of a nonoptimal response to imatinib as defined by the 2013 European LeukemiaNet (ELN) recommendations.^2^ The cutoff date for treatment monitoring was August 31, 2015. The study was approved by the Ethics Committee of Peking University People's Hospital, and all of the patients provided written informed consent to participate in the study in accordance with the Declaration of Helsinki.

**TABLE 1:**
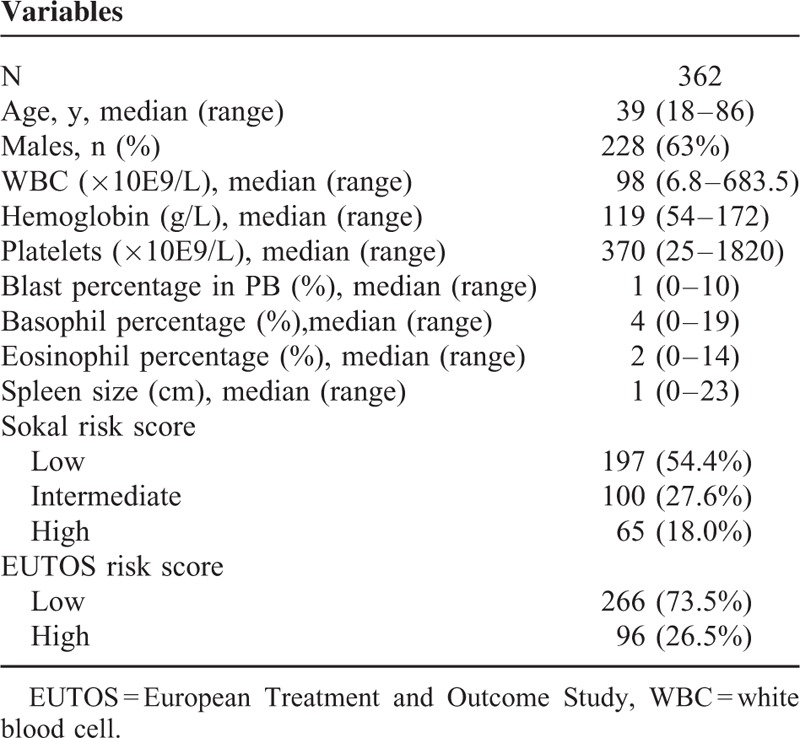
Characteristics of the Patients at Presentation

### Morphologic, Cytogenetic, and Molecular Analyses

Bone marrow (BM) aspirations for morphological and cytogenetic analyses were performed every 3 to 6 months until a complete cytogenetic response (CCyR) was achieved, and they were performed every 6 to 12 months thereafter. Cytogenetic analyses were performed with standard G-banding techniques, and for the follow-up analyses of CCyR, at least 20 metaphases were evaluated. The BCR-ABL transcript levels were detected at presentation, after 3 and 6 months of imatinib treatment, and every 3 to 6 months thereafter using peripheral blood (PB) or BM samples. As we previously reported,^30^ the BCR-ABL transcript levels were detected using the real-time quantitative polymerase chain reaction (RQ-PCR) method, and ABL was used as the control gene. The results were reported as BCR-ABL/ABL in percentage. All of the results used in this study presented ABL copies ≥ 32,000. The raw BCR-ABL transcript levels were converted to BCR-ABL^IS^ levels using our laboratory-specific conversion factor of 0.65, which was determined and validated through a sample exchange with Institute of Medical and Veterinary Science (IMVS) international reference laboratory in Adelaide, Australia.^31^ To generate comparable values, all of the BCR-ABL levels were converted to BCR-ABL^IS^ levels regardless of whether the value was < 10% after conversion. Confirmed major molecular response (MMR), MR4, and MR4.5 were defined as BCR-ABL^IS^ ≤0.1%, ≤0.01%, and 0.0032% or undetectable BCR-ABL, respectively, along with the presence of 10,000 to 32,000 and >32,000 ABL copies in 2 consecutive analyses.^11^ Hereafter, MMR, MR4, and MR4.5 refer to confirmed MMR, MR4, and MR4.5, respectively, and the time to achieve these responses was the month for which the first value was obtained for the corresponding confirmed responses.

### Treatment Efficacy Definitions and Statistical Analysis

The EFS, PFS, and OS values were calculated from the beginning of imatinib therapy to the last follow-up appointment or first event, with the event criteria listed as follows: for OS, the criterion was death from any cause; for PFS, the criteria were the beginning of accelerated phase (AP) or blast phase (BP) or death from any cause; and for EFS, the criteria included the criteria for PFS and a loss of complete hematologic or major cytogenetic response. Receiver operating characteristic (ROC) curves were used to demonstrate the association between achieving MR4.5 and WBC counts at presentation. The optimal thresholds along the ROC curves were calculated using the Youden index (sensitivity + specificity – 1). Cumulative incidences of responses were calculated by considering the competing risks defined by death. Comparisons between cumulative incidences were performed by the Gray test. Six patients who received allogeneic stem cell transplantation were censored at the time of transplantation in the calculation of cumulative incidence. Survival functions were estimated using the Kaplan–Meier method and compared among patients grouped according to their parameters at presentation using the log-rank test. Multivariate analyses were performed using the Fine-Gray model for cumulative incidence and the Cox proportional hazards model for survival to identify the independent prognostic variables. The parameters with *P* < 0.20 according to the univariate analysis were entered into a multivariate model. R version 2.6.1 (The R Foundation for Statistical Computing), SPSS 13.0 (SPSS Inc., Chicago, IL), and GraphPad Prism 5 software (GraphPad Software Inc., La Jolla, CA) were used.

## RESULTS

### Patient Characteristics, Responses, and Survival

Table 1 shows the characteristics of the patients at presentation in this study. A total of 18.0% and 26.5% of the patients belonged to the high-risk group according to the Sokal and EUTOS risk scores, respectively (Table 1). Patients belonging to the low and intermediate Sokal risk groups were all defined as the low Sokal risk group in this study according to 2013 ELN recommendations.^2^ The median follow-up time was 36 months (range 6–115). The 1-year cumulative incidences of CCyR and MMR were 87.4% (95% confidence interval [CI] 83.7–90.8%) and 49.1% (95% CI 43.3–53.8%), respectively. The cumulative incidences of MMR, MR4, and MR4.5 reached 89.3% (95% CI, 83.3–93.9%), 50.7% (95% CI, 43.1–57.3%), and 35.4% (95% CI, 42.1–56.3%) after 4 years, respectively. Overall, 15 patients progressed to advanced disease after treatment for a median of 12 months (range 3–36). Seven patients died, with all dying from disease progression. The 4-year EFS, PFS, and OS values were 91.6% (95% CI, 87.8–94.3%), 94.6% (95% CI, 91.2–96.8%), and 97.2% (95% CI, 93.1–98.7%), respectively.

### Patients With an Optimal Molecular Response to Imatinib in the First Year Had a Lower WBC Count at Presentation

**FIGURE 1:**
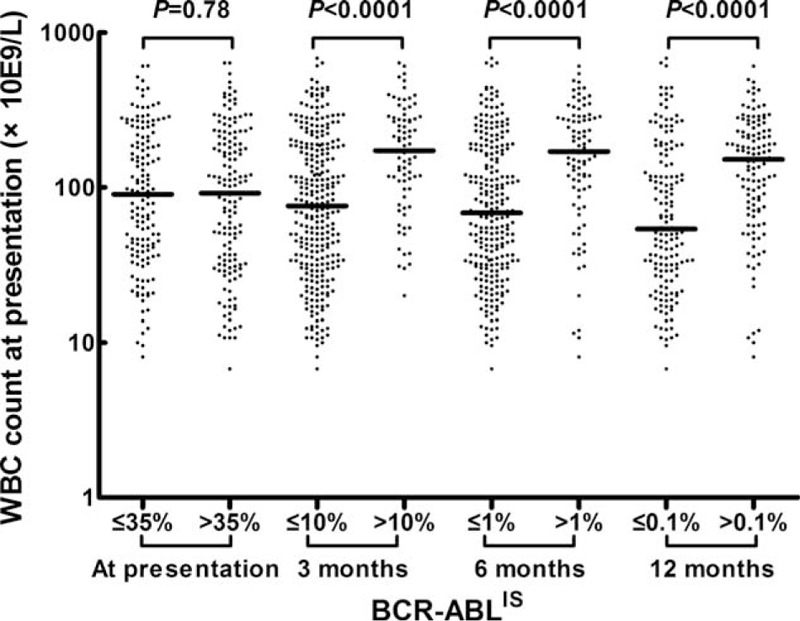
Comparisons of the WBC counts at presentation between patients grouped according to their BCR-ABL^IS^ at various time points. “−” represents the median value. BCR-ABL^IS^ = BCR-ABL transcript levels according to the international scale, WBC = white blood cell.

The patients were grouped according to their BCR-ABL^IS^ at various time points. The cutoff value used to group patients at presentation was their median levels, and these values at 3, 6, and 12 months were 10%, 1%, and 0.1%, respectively, which corresponded to the definition of optimal response according to the 2013 ELN recommendations.^2^ As shown in Figure 1, the WBC count had no impact on the BCR-ABL^IS^ at presentation (n = 293). Patients who showed an optimal response to imatinib at 3, 6, and 12 months had a significantly lower WBC count at presentation compared with those who exhibited a nonoptimal response (n = 362, 334 and 279, all *P* < 0.0001).

### Relationship Between WBC Count and Other Variables at Presentation

As shown in Table 2, the following factors were individually and significantly related to high WBC counts at presentation: age < 40 years, hemoglobin levels < 120 g/L, platelet counts ≤ 300 × 10E9/L, blast cells in PB, eosinophils in PB ≥ 5%, palpable spleen, high Sokal risk scores, and high EUTOS risk scores (all *P* < 0.05).

**TABLE 2:**
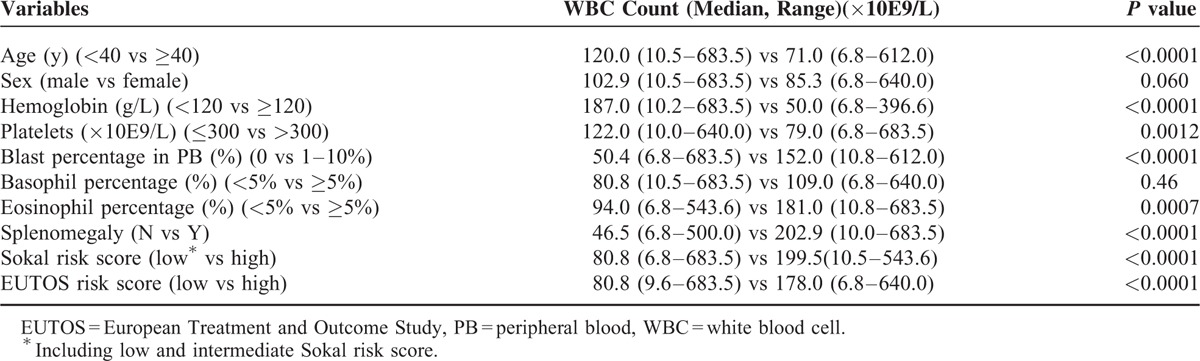
Relationship Between WBC Count and Other Variables at Presentation

### Determination of WBC Count Cutoff Level at Presentation

**FIGURE 2:**
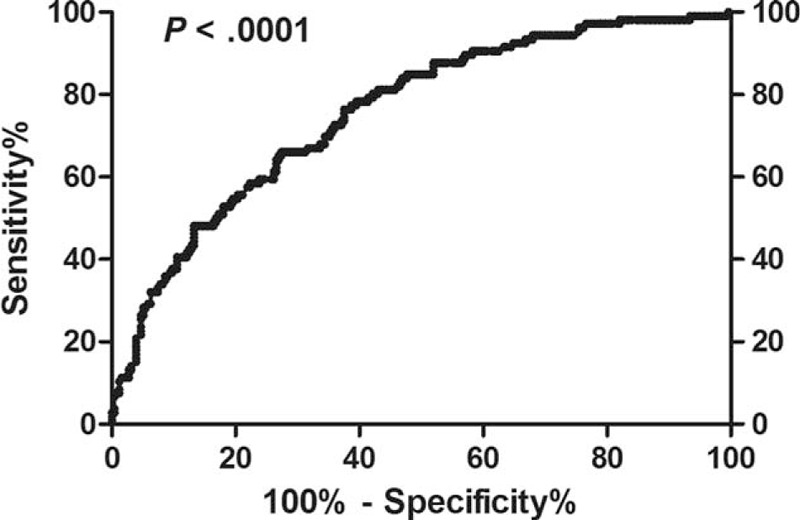
ROC curve of the relationship between WBC counts at presentation and achievement of a MR4.5. ROC = receiver operating characteristic, WBC = white blood cell.

The ROC curve revealed that the WBC count at presentation could significantly differentiate patients with different incidences of MR4.5 (area under the curve 0.76, *P* < 0.0001, Figure 2). In general, the sensitivity increased and the specificity decreased along with increases of WBC counts at presentation. Therefore, we calculated the Youden index for each cutoff value and found that it fluctuated within a small range (0.34–0.38) for cutoff values from 34 to 151 × 10E9/L. Because patients could be further differentiated according to their molecular response at 3 months, sensitivity (to identify patients who might achieve an MR4.5 as completely as possible) was more important than specificity (to identify patients who might achieve an MR4.5 as accurately as possible) at presentation. Therefore, 150 × 10E9/L was selected as the cutoff value for WBC counts at presentation in the subsequent analysis.

### Univariate and Multivariate Analysis of the Impact of Parameters on MR4.5 Achievement

**FIGURE 3:**
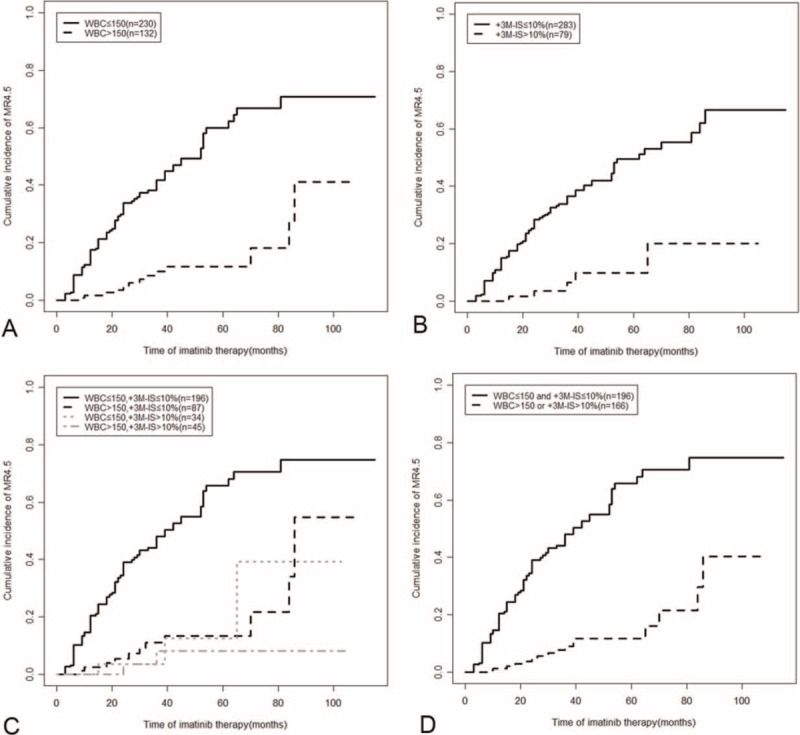
Cumulative incidence of MR4.5 among patients grouped according to their WBC counts at presentation and BCR-ABL^IS^ at 3 months. BCR-ABL^IS^ = BCR-ABL transcript levels according to the international scale, WBC = white blood cell.

The univariate analysis showed that following variables at presentation were all significantly related to a higher cumulative incidence of MR4.5: female sex, WBC count ≤ 150 × 10E9/L, hemoglobin ≥ 120 g/L, platelet count > 300 × 10E9/L, absence of blast cells in PB, and absence of a palpable spleen (Table 3, Figure 3A). Furthermore, patients with a low Sokal risk score had a significantly higher 4-year MR4.5 rate compared with those that had high risk scores, whereas the EUTOS risk score had no impact on the achievement of MR4.5 (Table 3). In addition, a BCR-ABL^IS^ value ≤ 10% at 3 months was also significantly related to the achievement of MR4.5 (Table 3, Figure 3B).

**TABLE 3:**
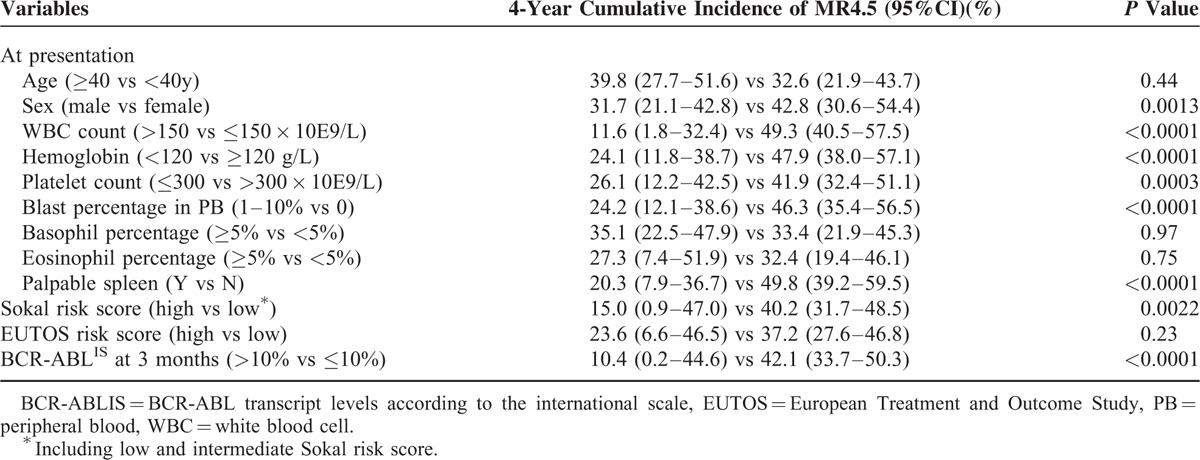
Univariate Analysis on the Achievement of MR4.5

We further performed 2 multivariate analyses. The first included the above-mentioned variables at presentation (risk scores were not included) and BCR-ABL^IS^ at 3 months (*P* < 0.20), and the second multivariate analysis included sex, WBC count, hemoglobin level, Sokal risk score, and BCR-ABL^IS^ at 3 months as parameters. As shown in Table 4, regardless of whether the Sokal risk score was included, the BCR-ABL^IS^ at 3 months, WBC count at presentation, hemoglobin levels, and sex were the common independent predictors for an MR4.5. PLT count at presentation was also an independent prognostic factor when the Sokal risk score was not involved, whereas the Sokal risk score was not an independent predictor for an MR4.5.

**TABLE 4:**
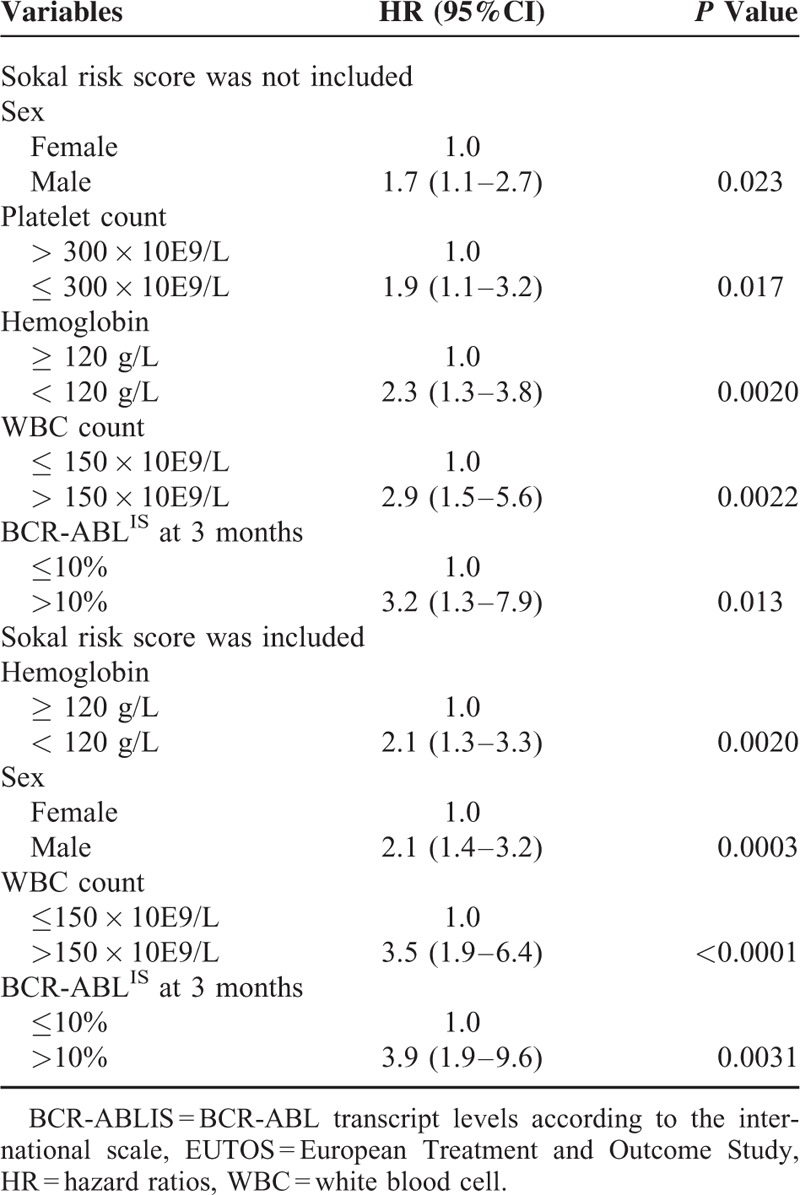
The Independent Prognostic Parameters on the Achievement of MR4.5

Among the independent prognostic factors, the BCR-ABL^IS^ at 3 months and WBC count at presentation were the parameters with the highest hazard ratio (HR, Table 4) in the 2 multivariate analyses, which indicates that they had greater impacts on achieving an MR4.5. Furthermore, they individually represented response indicators during treatment as well as patient biological characteristics. Therefore, we combined these factors to differentiate patients.

### Combination of BCR-ABL^IS^ at 3 Months and WBC Count at Presentation Better Predicted the Achievement of MR4.5 Compared With BCR-ABL^IS^ at 3 Months Alone

The patients were categorized into 4 groups according to their WBC count at presentation and BCR-ABL^IS^ at 3 months. As shown in Figure 3C, the patients with concurrent WBC counts at presentation of > 150 × 10E9/L (abbreviated WBC > 150) and BCR-ABL^IS^ at 3 months ≤ 10% (abbreviated BCR-ABL^IS^ ≤ 10%) (n *=* 87, 24%) exhibited a 4-year MR4.5 rate similar to that of patients with concurrent WBC counts ≤ 150 × 10E9 (abbreviated WBC ≤ 150) and BCR-ABL^IS^ at 3 months > 10% (abbreviated BCR-ABL^IS^ > 10%) (n *=* 34) and with concurrent WBC > 150 and BCR-ABL^IS^ > 10% (n *=* 45) (13.5% [95% CI 0–37.7%] vs 13.2% [95% CI 0–64.9%] vs 8.8% [95% CI 0–55.1%], *P* = 0.47), and these values were all significantly lower than that of patients with concurrent WBC ≤ 150 and BCR-ABL^IS^ ≤ 10% (n *=* 196) (55.0% [95% CI 46.5–62.7%], all *P* < 0.0001, Figure 3C). These results implied that BCR-ABL^IS^ at 3 months > 10% predicts a lower incidence of MR4.5, and WBC count at presentation > 150 × 10E9/L showed a similar predictive ability. Therefore, these 3 groups were merged into 1 group. As a result, patients with WBC > 150 or BCR-ABL^IS^ > 10% (n *=* 166, 45.9%) had significantly lower 4-year MR4.5 rates compared with those with concurrent WBC ≤ 150 and BCR-ABL^IS^ ≤ 10% (n *=* 196, 54.1%) (12.0% [95% CI 2.1–31.0%] vs 55.0% [95% CI 46.5–62.7%], *P* < 0.0001, Figure 3D). The combination of BCR-ABL^IS^ at 3 months and WBC count at presentation accurately identified another 24% (87/362) of patients as poor deep molecular responders to imatinib compared with BCR-ABL^IS^ at 3 months alone.

### Survival Analysis

**FIGURE 4:**
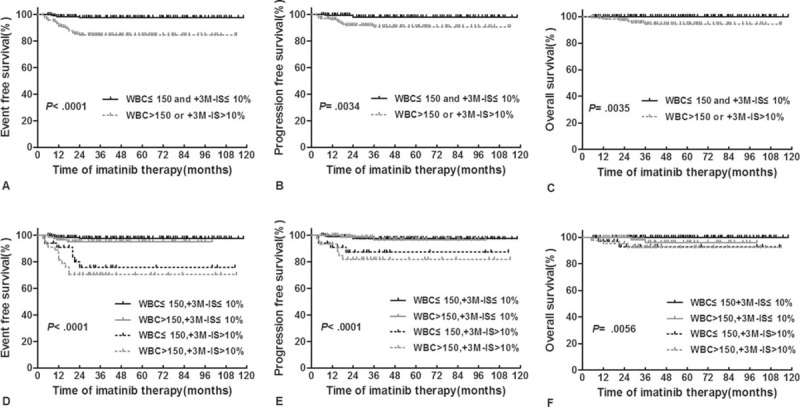
Survival analysis of patients grouped according to their WBC counts at presentation and BCR-ABL^IS^ at 3 months. (A–C) Patients were grouped into 2 groups; (D–F) patients were grouped into 4 groups. BCR-ABL^IS^ = BCR-ABL transcript levels according to the international scale, EFS = event-free survival, OS = overall survival, PFS = progression-free survival, WBC = white blood cell.

The patients with concurrent WBC ≤ 150 and BCR-ABL^IS^ ≤ 10% had better 4-year EFS, PFS, and OS compared with those presenting WBC > 150 or BCR-ABL^IS^ > 10% (EFS: 97.7% [95% CI 93.9–99.1%] vs 84.6% [95% CI 77.6–89.5%], *P* < 0.0001; PFS: 98.0% [95% CI 93.9–99.3%] vs 90.8% [95% CI 84.5–94.6%], *P* = 0.0034; OS: 100% vs 94.4%[95% CI 88.4–97.4%], *P* = 0.0035, Figure 4A–C).

We performed further comparisons between the 4 groups (Figure 4D–F). For the 4-year EFS and PFS rates, patients with concurrent WBC ≤ 150 and BCR-ABL^IS^ ≤ 10% had similar values to those with concurrent WBC > 150 and BCR-ABL^IS^ ≤ 10% (EFS: 97.7% vs 95.1%, PFS: 97.5% vs 96.5%, all *P* > 0.05). The patients with concurrent WBC ≤ 150 and BCR-ABL^IS^ > 10% had similar values to those with concurrent WBC > 150 and BCR-ABL^IS^ > 10% (EFS: 75.9% vs 70.4%, PFS: 87.3% vs 82.0%, all *P*> 0.05), and the former 2 groups presented significantly higher values than the latter two (EFS: *P* ≤  0.0064, PFS: *P* ≤  0.026). For the 4-year OS rates, the patients with concurrent WBC > 150 and BCR-ABL^IS^ ≤ 10% presented similar to those with concurrent WBC ≤ 150 and BCR-ABL^IS^ > 10% and with concurrent WBC > 150 and BCR-ABL^IS^ > 10% (96.2% vs 93.0% vs 92.5%, *P* > 0.05), and the values were all significantly lower than those of patients with concurrent WBC ≤ 150 and BCR-ABL^IS^ ≤ 10% (100%, *P* = 0.032, 0.0006, and 0.0003). Therefore, patients with concurrent WBC ≤ 150 and BCR-ABL^IS^ ≤ 10% exhibited significantly lower event and progression risks compared with those with BCR-ABL^IS^ > 10% and significantly lower death risks compared with those with WBC > 150 or BCR-ABL^IS^ > 10%.

## DISCUSSION

In the current TKI era, rapidly achieving a deep molecular response is the advanced treatment goal in CML-CP patients.^6,7^ In addition to EMR, baseline prognostic predictions should be performed to ensure that patients receive the most appropriate initiative TKI (imatinib or a second-generation TKI). In this study, we found that the optimal molecular response in the 1st year was related to low WBC counts at presentation, and a BCR-ABL^IS^ at 3 months > 10% and WBC count > 150 × 10E9/L at presentation were independently poor prognostic factors for MR4.5. Furthermore, patients with concurrent WBC > 150 and BCR-ABL^IS^ ≤ 10% had a similarly low incidence of MR4.5 as patients with BCR-ABL^IS^ > 10%, whereas patients with concurrent WBC ≤ 150 and BCR-ABL^IS^ ≤ 10% presented a significantly higher incidence of MR4.5 and better survival. Therefore, a combination of the molecular response at 3 months with the WBC count at presentation provided accurate predictions of the deep molecular response of imatinib-treated CML-CP patients. The WBC count at presentation might be used to differentiate patients at the beginning of imatinib treatment.

Most CML-CP patients quickly achieve CCyR with imatinib treatment, and their subsequent leukemic burden is only accurately reflected by the BCR-ABL transcript levels as detected by RQ-PCR. The use of BCR-ABL^IS^ enables a comparison of results from different laboratories.^32^ We previously reported that the early cytogenetic and molecular responses were similar between our study and the Germany CML-Study IV.^31^ Additionally, the differences in the definition of a molecular response and the analyzed cohorts resulted in different reported molecular response rates.^11,19^ Here, we followed the definition used in the CML-Study IV trial, and the 4-year cumulative rates were comparable between our study (MR4, 50.7%; MR4.5, 35.4%) and their results (MR4, 60.4%; MR4.5, 41.9%). This result indicates that our deep molecular response evaluation was appropriate. The use of stricter definitions might have caused the lower MR4 and MR4.5 rates reported by the IMVS laboratory in Adelaide.^19^

Currently, risk scores, such as the Sokal or EUTOS scores, are usually used to stratify patients; however, they have not been established for deep molecular response predictions.^21–23^ A high WBC count at diagnosis is an important negative prognostic pretreatment parameter for acute leukemia,^28,29^ whereas its prognostic value in CML has remained unclear throughout the past decade of TKI use. In this study, we provided the first observations that the degree of molecular response at 3 time points in the first year of treatment is significantly related to the WBC count at presentation and showed that a higher WBC count is associated with a worse molecular response. The ROC curve indicated that WBC counts are significantly related to the MR4.5. To identify the greatest amount of patients who might achieve an MR4.5, a cutoff value of 150 × 10E9/L was selected to group patients who had values with a similar Youden index.

We analyzed the impact of parameters at presentation and BCR-ABL^IS^ at 3 months on the achievement of MR4.5. Similar to the report by Branford et al, sex but not age was a prognostic indicator for deep molecular response.^19^ Recently, Lin et al reported that sex and BCR-ABL type but not age were significantly associated with MMR achievement.^33^ Spleen size was incorporated into all 3 risk scores,^21–23^ and we also found that this parameter was related to MR4.5. Our group and other researchers have previously reported that high baseline hemoglobin is correlated with a higher likelihood of achieving 3-month CCyR and survival in second-generation TKI-treated imatinib-resistant CP patients and AP patients treated with both imatinib and allogeneic hematopoietic stem cell transplantation.^34,35^ However, hemoglobin has not been analyzed with regard to its ability to predict deep molecular responses. Similar to the results of other reports, the Sokal risk score was found to be related the MR4.5, whereas the EUTOS risk score had no impact on the MR4.5.^24–26,36–38^ The basophil percentage has been recognized as a signal of CML progression,^39^ and it was included in the EUTOS risk score;^23^ however, the mechanism of its prognostic impact remains unknown. Hughes et al reported that the number of basophils was relevant to the 12-month but not the 24-month MMR rate in patients receiving 600 mg/day of imatinib.^40^ Here, we could not identify the impact of basophil number on the MR4.5. A BCR-ABL^IS^ at 3 months > 10% has been correlated with a poor outcome and cytogenetic and molecular responses in a number of studies.^16–18^ As a result, this value is defined as a treatment failure by the National Comprehensive Cancer Network (NCCN) guidelines and provided as a warning by the ELN recommendations. The prognostic value of the BCR-ABL^IS^ at 3 months to the achievement of an MR4.5 was demonstrated in this study.

Whether the Sokal risk score was included, the sex, hemoglobin level, WBC count, and BCR-ABL^IS^ at 3 months were the common independent predictors for a MR4.5. Although the identified independent predictors were related to the involved factors, our conclusions on the prognostic value of sex and BCR-ABL^IS^ at 3 months were the same as that of Branford et al,^19^ which implied that these predictors have important roles in the achievement of an MR4.5. Furthermore, the Sokal or EUTOS risk scores were not found to be independent prognostic of an MR4.5. During the establishment of the Sokal risk score, the WBC count was found to be insignificant in the multivariable regression. This discrepancy implied that the predictors for survival and deep molecular response might not be completely equivalent and suggests that TKI treatment may have an impact on predictors. Therefore, the predictor for deep molecular response must be comprehensively investigated in relation to TKI usage. In addition, we found that several variables were significantly related to the WBC count at presentation. The significant parameters in the univariate analysis, such as the blast percentage in PB, palpable spleen, and Sokal and EUTOS risk scores, were not shown to be independent predictors, which indicated that the WBC count is a more important predictor and counteracts their effects on the achievement of an MR4.5.

The baseline parameters and EMR individually represents certain patient characteristics and provides real responses to treatment. Recently, Tiribelli et al showed that a combination of the EUTOS Score and BCR-ABL^IS^ at 3 months identified a group of CML patients with a favorable response to imatinib, suggesting that a combination of independent parameters might better stratify patients.^27^ Therefore, we combined the WBC count at presentation and BCR-ABL^IS^ at 3 months, which were the independent predictors with the highest HR. Moreover, we showed for the first time that patients with BCR-ABL^IS^ ≤ 10% but WBC count > 150 (24% of all patients) had similarly low incidences of MR4.5 compared with those with BCR-ABL^IS^ > 10%. Therefore, both BCR-ABL^IS^ at 3 months > 10% and WBC count at presentation > 150 predicted a poor deep molecular response to imatinib. The introduction of WBC count at presentation improved the ability to predict an MR4.5.

The primary treatment goal is survival. We found that patients with concurrent WBC ≤ 150 and BCR-ABL^IS^ ≤ 10% had significantly better outcomes than those with WBC > 150 or BCR-ABL^IS^ > 10% and significantly higher survival rates than those in each of the other 3 groups. Therefore, by combining WBC count at presentation with BCR-ABL^IS^ at 3 months, we could differentiate a group of patients that presented a significantly higher incidence of MR4.5 and better outcome.

Why does WBC count affect the achievement of MR4.5? We hypothesized that the WBC count at presentation might represent the duration between the initial occurrence to diagnosis of CML. CML patients usually present genetic instability,^41,42^ and the longer the disease duration, the greater the number of genetic abnormalities they acquire and the higher the percentage of drug-resistant cells they might present.

This study had certain limitations. First, it was a retrospective analysis and all patients had available BCR-ABL^IS^ results at 3 months, whereas the BCR-ABL^IS^ results were incomplete at presentation, 6 and 12 months. Second, the samples used for the molecular detection included PB and BM. BM was the main sample source for the RQ-PCR during the early stage of imatinib application for CML at our center. Subsequently, PB has been increasingly accepted, and it has become the primary sample source. Therefore, a prospective multicenter trial is warranted to confirm the results of this study.

In conclusion, both WBC at presentation ≤ 150 × 10E9/L and BCR-ABL^IS^ at 3 months ≤ 10% were independently associated with the achievement of an MR4.5 in imatinib treated CML-CP patients. Patients with concurrent WBC > 150 and BCR-ABL^IS^ ≤ 10% showed a similarly low incidence of MR4.5 compared with those with BCR-ABL^IS^ > 10%. A combination of molecular response at 3 months with WBC count at presentation better stratified imatinib-treated CML-CP patients with regard to their deep molecular response and outcome. Whether a second-generation TKI could improve the responses and outcomes of patients with WBC > 150 remains to be evaluated. The conclusions presented here might allow CML-CP patients to receive more accurate and effective treatment as early as possible so that they quickly achieve an MR4.5 and prepare for TKI discontinuation.
